# Effect of locally delivered adjunctive antibiotics during surgical periodontal therapy: a systematic review and meta-analysis

**DOI:** 10.1007/s00784-021-04056-7

**Published:** 2021-07-20

**Authors:** Sarah Yusri, Ahmed Elfana, Weam Elbattawy, Karim M Fawzy El-Sayed

**Affiliations:** 1 Oral Medicine and Periodontology Department, Faculty of Dentistry, Cairo University, Al Saraya Str. 11, Manial, Cairo, Egypt; 2 Clinic for Conservative Dentistry and Periodontology, School of Dental Medicine, Christian Albrechts University, Kiel, Germany

**Keywords:** Local, Anti-bacterial, Antibiotics, Surgical, Periodontal therapy, Systematic review

## Abstract

**Aim:**

The present study aimed to systematically assess current evidence on effects of locally delivered antibiotics during periodontal surgery compared to periodontal surgery alone on clinical attachment level (CAL) gain, probing pocket depth (PPD) reduction, recession depth (RD) changes, gingival index (GI), bleeding on probing (BOP), and plaque index (PI).

**Methodology:**

MEDLINE-PubMed, Cochrane-CENTRAL and Scopus databases were searched up to April 2021 for randomized clinical trials (RCT), evaluating effects of locally delivered antibiotics during periodontal surgery. CAL gain served as primary, while PPD reduction, RD changes, GI and PI as secondary outcomes. The Cochrane Risk of Bias Tool was used to assess possible bias. Data were extracted, and meta-analysis was performed where appropriate.

**Result:**

Screening of 2314 papers resulted in nine eligible studies. No adverse events were reported. Data on outcome variables were pooled and analyzed using generic inverse variance model and presented as weighted mean difference (WMD) and 95% confidence interval (95% CI). Statistically significant improvements in favor of antibiotics’ delivery were observed in studies with follow-up of ≤6 months for CAL gain (WMD = 0.61 mm (95% CI [0.07, 1.14]; *p* = 0.03), PPD reduction (WMD = 0.41 mm (95% CI [0.02, 0.80]; *p* = 0.04)) and BOP (WMD = −28.47% (95% CI [−33.00, −23.94]); *p* < 0.001), while for GI improvements were notable for >6 to 12 months (WMD = −0.27 (95% CI [−0.49, −0.06]; *p* = 0.01)).

**Conclusion:**

Within the current review’s limitations, locally delivered antibiotics during surgical periodontal therapy results in post-surgical improvements for CAL, PPD, and BOP (≤6 months) with a longer-lasting GI improvement. Further randomized controlled trials are needed with true periodontal end-points to assess the ideal antibiotic agent, dosage, and delivery methods.

**Clinical relevance:**

Local delivery of antibiotics during periodontal surgery improved clinical parameters for up to 6-month follow-up, with beneficial longer effects on gingival inflammation. Within the current study’s limitation, the presented evidence could support the elective usage of locally delivered antibiotics during surgical periodontal therapy.

**Supplementary Information:**

The online version contains supplementary material available at 10.1007/s00784-021-04056-7.

## Introduction

Periodontitis is an inflammatory destructive disorder of the tooth-supporting and -investing structures, associated with microbial dysbiosis [1]. Reversing periodontitis-associated dysbiosis is one of the primary strategies of periodontal therapy, comprising clinical strategies, implying the improvement of patients’ self-performed oral hygiene [2], in combination with professional mechanical debridement [3, 4] and in selective clinical conditions the application of adjunctive antimicrobial agents, systemically [5–7] or locally [8, 9]. Compared to systemic antibiotic application, local delivery demonstrates lower incidence of side effects, improved compliance, with lesser chances for emergence of microbial resistance [9, 10].

Although professional non-surgical periodontal debridement remains the cornerstone of successful periodontal therapy, certain clinical conditions may necessitate the application of surgical approaches [11, 12]. In this context, a controversy exists regarding a possible effect of adjunctive systemic antimicrobials combined with subgingival plaque control on healing conditions and subsequent clinical outcomes of surgical periodontal therapy [13, 14]. Despite the possible benefits of locally delivered antibiotics in conjunction with surgical periodontal therapy, currently no clear evidence exists for their usage.

Thus, the objective of the present study was to systematically compile and analyze data from human studies on locally delivered adjunctive antibiotic therapy during periodontal surgery. The focused PICOS question was: Based on randomized controlled clinical trials (RCTs) on humans, what is the effect of locally delivered adjunctive anti-bacterial therapy during periodontal flap surgery, treating periodontitis-induced defects, compared to periodontal surgery alone, on clinical attachment level (CAL; primary outcome) gain, the probing pocket depth (PPD) reduction, the gingival recession depth (RD) changes, bleeding on probing (BOP), gingival index (GI), plaque index (PI) and radiographic changes (secondary outcomes)? We further aimed to perform a meta-analysis for the findings from different studies and to appraise their validity and applicability.

## Materials and methods

### Protocol registration and focused question

The protocol of this review article was pre-registered at the Prospective Register of Systematic Reviews (PROSPERO) on the 16th of February 2021 (CRD42021227099). This systematic review was conducted in accordance with the Cochrane handbook for systematic reviews of interventions [15] and reported in accordance with “Preferred Reporting Items for Systematic Review and Meta-Analysis” (PRISMA) guidelines [16]. The review aimed to answer the following question: During surgical intervention for treatment of periodontal diseases, will locally delivered antibiotics yield superior effects compared to periodontal surgery alone?

### Search strategy

Primary search was conducted in electronic sources of the National Library of Medicine (MEDLINE via PubMed), the Cochrane Central Register of Controlled Trials (CENTRAL), and Scopus. Other sources for search included clinical trials registry (http://www.clinicaltrials.gov/) and grey literature sources (Open-grey: http://www.opengrey.eu/; Grey literature report: http://www.greylit.org/). Reference lists of initially selected studies were hand-searched for further published work that could meet the eligibility criteria. In addition a forward search of the citing literature reported in Google scholar citation report was conducted. The search was conducted up to the 15th of April 2021. The search strategy employed an approach, joining keywords for local antibiotics with keywords for periodontal surgery (Appendix 1), without language restriction.

### Screening and selection

All entries from search databases were pooled into a single list and duplicates were removed (Endnote X9, Clarivate Analytics, USA), then exported to Rayyan online tool [17]. Two authors (SY, AE) independently conducted the initial screening of titles and abstracts, according to the eligibility criteria. Studies were selected for further reading if their title and/or abstract presented suitability to eligibility aspects. Disagreement between the two reviewers was resolved after additional discussion, and if it persisted, judgment by a third author (KFE) was conclusive. After initial selection, full-text papers were read in detail (SY, AE) and in case of unresolved disagreement, arbitration was sought after (KFE). Reasons for articles’ exclusion were recorded, and papers that fulfilled all selection criteria were processed for data collection. Cohen’s kappa coefficient was used to calculate agreement between authors [18].

The following eligibility criteria were employed:

(I) Study design: Randomized controlled clinical trials (RCTs) applying locally delivered antibiotics during periodontal flap surgery, treating periodontitis-induced defects. Studies should have quantitatively appraised one or more of the predefined outcomes (see below).
(II) Population: Adult participants diagnosed with periodontitis and undergoing periodontal surgery.
(III) Intervention: Surgical periodontal intervention of sites with PPD ≥5 mm in conjunction with locally delivered antibiotics into the surgical defects at the time of surgery.
(IV) Control: Surgical periodontal intervention without locally delivered antibiotics.
(V) Outcomes: Seven outcomes were assessed: CAL gain (the distance measured from the cementoenamel junction (CEJ) to the depth of the periodontal pocket; primary outcome), PPD reduction (the distance measured from the free gingival margin to the depth of the periodontal pocket), RD changes (the distance measured from the CEJ to the free gingival margin), BOP [19], GI, and PI [20], and radiographic changes (secondary outcomes).

The following was set as exclusion criteria:

- Studies with unbalanced interventions and controls (e.g., applying bone grafts with antibiotics in the test-group and no bone graft nor antibiotics were applied in the control-group). Standard delivery vehicles of antibiotics (e.g., methylcellulose, carboxymethyl cellulose, carboxyethyl cellulose, polypropylene) were exempted.
- Studies on smokers and diabetic patients
- Non-periodontitis conditions (e.g., peri-implantitis, muco-gingival correction, extraction sockets)
- Studies reporting the use of pre-operative or post-operative systemic antibiotics
- Antibiotics used as topical application/mouthwash and not delivered directly into the surgical site
- Antibiotics used transiently during the surgery for root conditioning and/or rinsed after application prior to flap closure

### Quality assessment

The quality of the included studies was assessed using the Cochrane Risk of Bias Tool for randomized clinical trials (RoB 2, updated in 2019) [21] by AE and SY. Each study was judged as at low risk, high risk, or reflecting some concerns according to the following domains:

1. Randomization process: Allocation sequence generation, allocation concealment, and implementation
2. Deviations from intended interventions: Assessing both effect of assignment to intervention and effect of adhering to intervention
3. Missing outcome data: Availability of data for all or nearly all participants
4. Measurement of the outcome: Assessing method of measurement and whether prior knowledge of assigned group would influence outcome data.
5. Selection of the reported result: Data analysis is in accordance with pre-specified study plan.

For each study, the overall judgment was low risk in case of achieving low risk in all domains, unclear risk in case one or more domains demonstrated unclear risk, and high risk of bias when having at least one high risk domain or multiple unclear risk domains.

### Data extraction and study characteristics assessment

All included studies went through relevant data extraction through standardized pre-defined data extraction sheets (AE) and cross-checked independently by another author (SY). Data of interest were study design, number of defects/participants in each arm, study setting, the treated periodontal condition, type of interventions, type, form, and concentration of antibiotics used, follow-up intervals, reported systemic antibiotic embargo period, in addition to the pre-defined primary and secondary outcomes on basis of intention-to-treat analyses [22]. Authors were contacted for papers with missing data (AE).

### Quantitative synthesis

Studies were initially summarized for main characteristics and types of outcomes measured. Choice to pool results into meta-analysis was taken when two or more studies presented the same measured outcome. Outcomes’ data were recorded on patient’s level and were grouped into two effect durations: up to 6 months (≤6 months) and longer than 6 months (>6 to 12 months) [23, 24]. If the SD of the mean difference could not be retrieved it was imputed, using the Follmann method [25] from baseline and final values data, and an average correlation calculated from included studies [26]. In case this was not feasible, the average of other studies’ SD in the respective group was used [27]. For split-mouth studies, outcomes data were pooled, taking into account the within-person correlation by using results from paired tests or calculated from individual patients data (IPD) and if not available, SD was imputed with relevant correlation [26]. Summary of data synthesis is presented in Appendix 2.

The meta-analyses were conducted when feasible with the inverse variance model using Review Manager (RevMan, Version 5.4.1, The Cochrane Collaboration, 2020). Sub-analyses were performed on the observation periods up to 6 months and more than 6 months of follow-ups. Weighted averages of treatment effects across studies were calculated, using the random-effect model. In cases where the estimate of between study variance was poor, as a result of low number of pooled studies (when four or fewer studies were included) the fixed-effect analysis was used [28]. Forest plots were generated to visually present the treatment effects using weighted mean difference (WMD) and 95% confidence interval (95% CI) and statistical significance cutoff was set at *p* < 0.05.

### Assessment of heterogeneity

Heterogeneity was assessed statistically using Cochran’s Q and *I*^2^-statistics [29], and interpreted as low heterogeneity for *I*^2^ = 0–40%, moderate for *I*^2^ = 30–60%, substantial for *I*^2^ = 50–90% and considerable for *I*^2^ = 75–100% [30]. Publication bias was planned to be investigated with funnel plot and Egger regression intercept test [31], in case of 10 or more included studies in a meta-analysis. Sensitivity analyses were carried out assuming within-person correlation of 0, being the most conservative estimate [26], in addition to catering for variations in study designs (split-mouth and parallel groups).

### Strength of the evidence

The Grading of Recommendations Assessment, Development and Evaluation (GRADE) is a system to grade evidence quality and recommendation strength for data on a specific intervention [32]. The strength of evidence for the primary outcome (CAL gain) at follow-up >6 to 12 months was presented using the GRADE approach for grading the quality of evidence and the strength of our recommendations into very low, low, moderate, or high levels of evidence quality/certainty.

## Results

### Literature search results

**Figure 1:**
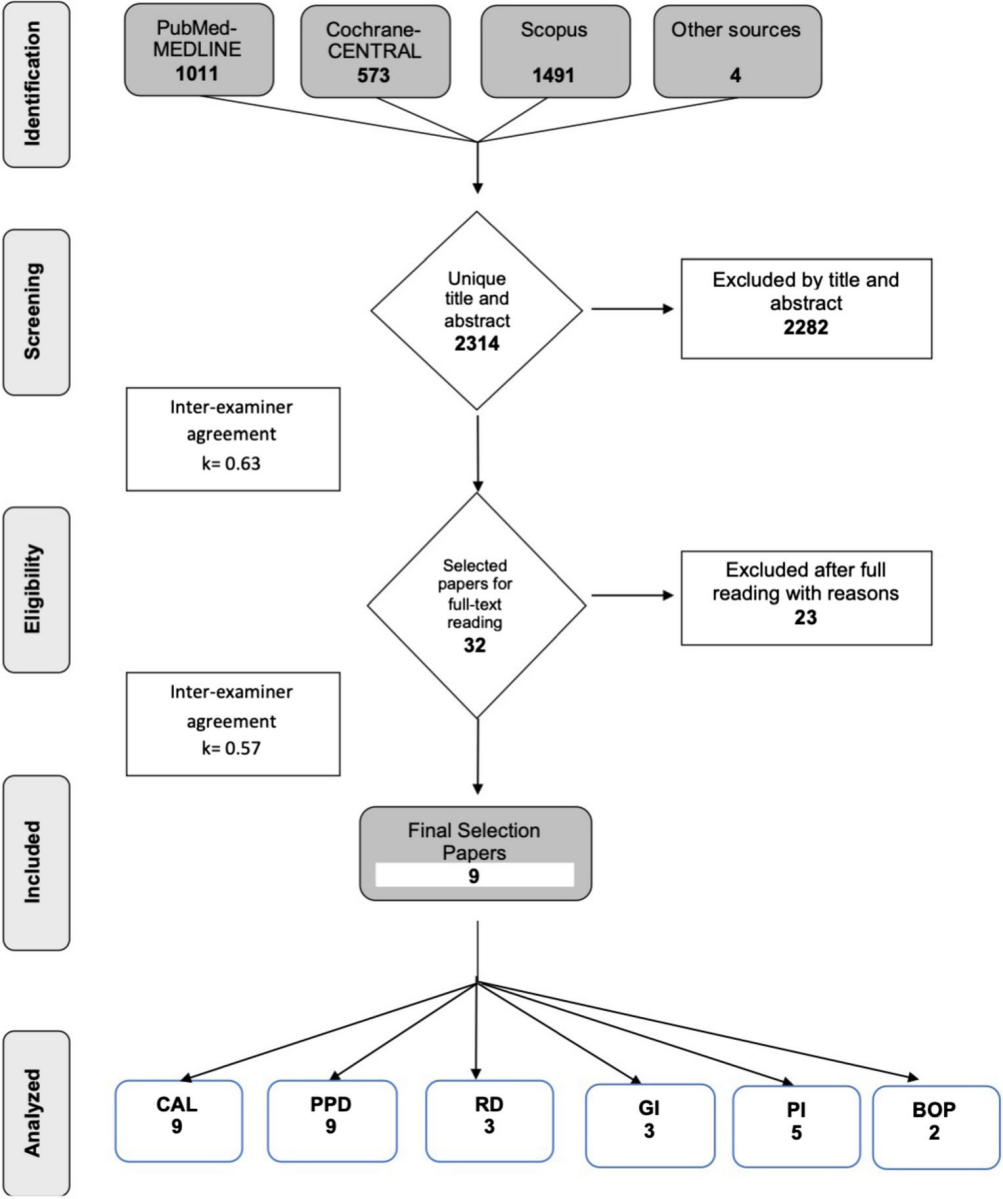
Study search and selection results

The online search yielded 1011 records in MEDLINE, 573 in Cochrane, 1491 in Scopus and four records were retrieved from other sources. After duplicate removal, a total of 2314 articles were screened for title and abstract, out of which 32 articles were selected for full text assessment (agreement *k* = 0.63). Thereafter, 23 articles were excluded (Figure 1; Appendix 3) and nine articles were included in this review (agreement *k* = 0.57).

### Included studies’ characteristics

Regarding the included RCTs, two studies had parallel groups [33, 34], while seven employed a split-mouth design [35–41]. Overall, the number of participants in the included data ranged from nine to 30 individuals per group and with a follow-up period ranging between 3 and 12 months. All studies randomized participants to the treatment groups, with blinding being reported only in five studies [33, 34, 36, 37, 39]. Investigations were conducted under university settings except in three studies where it was not reported [34, 38, 39]. Except for a single investigation [41], all studies reported no previous antibiotic use for the participants for the last 3 [34, 37, 38], 6 months [33, 39, 40], or generally [35, 36].

The periodontal condition requiring surgical treatment was defined as PPD≥6 mm in five studies [34–37, 39], PPD>6 mm [40], PPD>5 mm [38], having a mandibular furcation grade-II defects [33], or PPD ranging from 5 to 7 mm [41]. All studies reported the implementation of non-surgical periodontal therapy pre-operatively. Regarding the surgical interventions for osseous defect therapy, different approaches were employed, including open flap debridement (OFD) [35, 37], OFD with platelet-rich fibrin [41], modified Widman flap [34], guided tissue regeneration (GTR) using membrane [36, 40], GTR using membrane and bone graft [33] or bone grafts only [38, 39]. The most frequently locally delivered antibiotic agent was Metronidazole [34, 36, 40, 41], followed by Minocycline [35, 37], Doxycycline [33], Tetracycline [38], and Moxifloxacin [39]. Studies’ characteristics are summarized in Table 1.

**Table 1:** Overview of the included studies and characteristics processed for data extraction

**Study**	**Study design/randomization/blinding**	**Number of participants**	**Age**	**Gender (male/female)**	**Periodontal condition**	**Interventions**	**Antibiotic type (concentration)**	**Delivery method**	**Duration of follow-up**	**Study setting**	**Previous antibiotic use**
**Abbas et al.** **[**35**]**	Split-mouth/yes/not reported	30	30–50	Not mentioned	PPD≥6 mm, generalized chronic periodontitis	OFD with/without antibiotic	Minocycline 1 mg (2%)	Periofeel dental ointment	6 months^*^	University	No previous antibiotic use
**Dowell et al.** **[**36**]**	Split-mouth/yes/yes	16	43.7 ± 0.4	7/9	PPD≥6 mm and evidence of radiographic bone loss	GTR (membrane) with/without antibiotic	Metronidazole, 8 mg per 1 cm^2^	Collagen membrane	26 weeks	University	No previous antibiotic use
**Jung et al.** **[**37**]**	Split-mouth/yes/yes	20	43–69	14/6	At least 6 mm deep accompanying BOP. Chronic severe periodontitis	OFD with/without antibiotic	Minocycline 1 mg (2%)	Periocline ointment	6 months*	University	No previous antibiotic use in last 3 months
**Lyons et al.** **[**33**]**	Parallel/yes/yes	18	43–67		Moderate or severe chronic periodontitis and a mandibular molar with a buccal or lingual degree-II furcation defect	GTR (bone + membrane) with/without antibiotic	4% doxycycline hyclate	Poly (DL-lactide) PLA barrier	9 months	University	No previous antibiotic use in last 6 months
**Masters et al.** **[**38**]**	Split-mouth/yes/not reported	15	35–61	3/12	PPD>5 mm and radiographic evidence of an intrabony defect in patients with jadvanced adult periodontitis	Bone graft with/without antibiotic	Tetracycline hydrochloride, 50 mg/ml solution	DFDBA	12 months	Not mentioned	No systemic tetracycline or metronidazole therapy within 3 months of the surgery
**Needleman et al.** **[**34**]**	Parallel/yes/yes	38	35–65	Not mentioned	PPD≥ 6 mm with BOP, evidence of radiographic bone loss in moderate to advanced periodontitis	MWF surgery with/without antibiotic	25% metronidazole	Elyzol Dental Gel	12 months	Not mentioned	No systemic or local antimicrobial therapy in the previous 3 months
**Reddy et al.** **[**39**]**	Split-mouth/yes/yes	15	Not mentioned	Not mentioned	PPD of ≥6 mm, with radiographic evidence of vertical/angular defects. Chronic generalized periodontitis	Bone graft with/without antibiotic	Moxifloxacin 0.4%	Gel with inert matrix then mixed with hydroxy-apatite composite graft	12 months	Not mentioned	Not taken antibiotics within last 6 months
**Sander et al.** **[**40**]**	Split-mouth/yes/not reported	12	20–60	5/7	PPD >6 mm and evidence of radiographic 2 or 3-walled defect depth of 4 mm	GTR (membrane) with/without antibiotic	25% metronidazole 1 g	Paroject gel	6 months	University	No systemic antibiotics 6 months prior to the study
**Taneja et al.** **[**41**]**	Split-mouth/yes/not reported	20	35–55	Not mentioned	PPD in the range of 5–7 mm, radiographic evidence of vertical bone loss	(OFD + PRF) with/without antibiotic	Metronidazole 1%	Gel with inert matrix then mixed with PRF	9 months	University	Not mentioned

*BOP* bleeding on probing, *DFDBA* demineralized freeze-dried bone allograft, *GTR* guided tissue regeneration, *MWF* modified Widman flap, *OFD* open flap debridement, *PPD* probing pocket depth, *PRF* platelet-rich fibrin

^*^Only 3 months of data were used due to the application of topical antibiotics in the test group after 3 months

### Adverse effects and patient-reported outcomes during healing

None of the studies reported any adverse effects linked to antibiotic usage, with uneventful healing observed. A study reported that one participant experienced pain in site with antibiotic administration, while five other participants demonstrated pain and unpleasant taste in the control sites 1 week post-operatively, with no differences observed in healing [36]. The same study further reported that one of the treated-furcation defects of the control-group failed to close at the end of the follow-up period, and the tooth was deemed for extraction.

### Quality assessment

**Figure 2:**
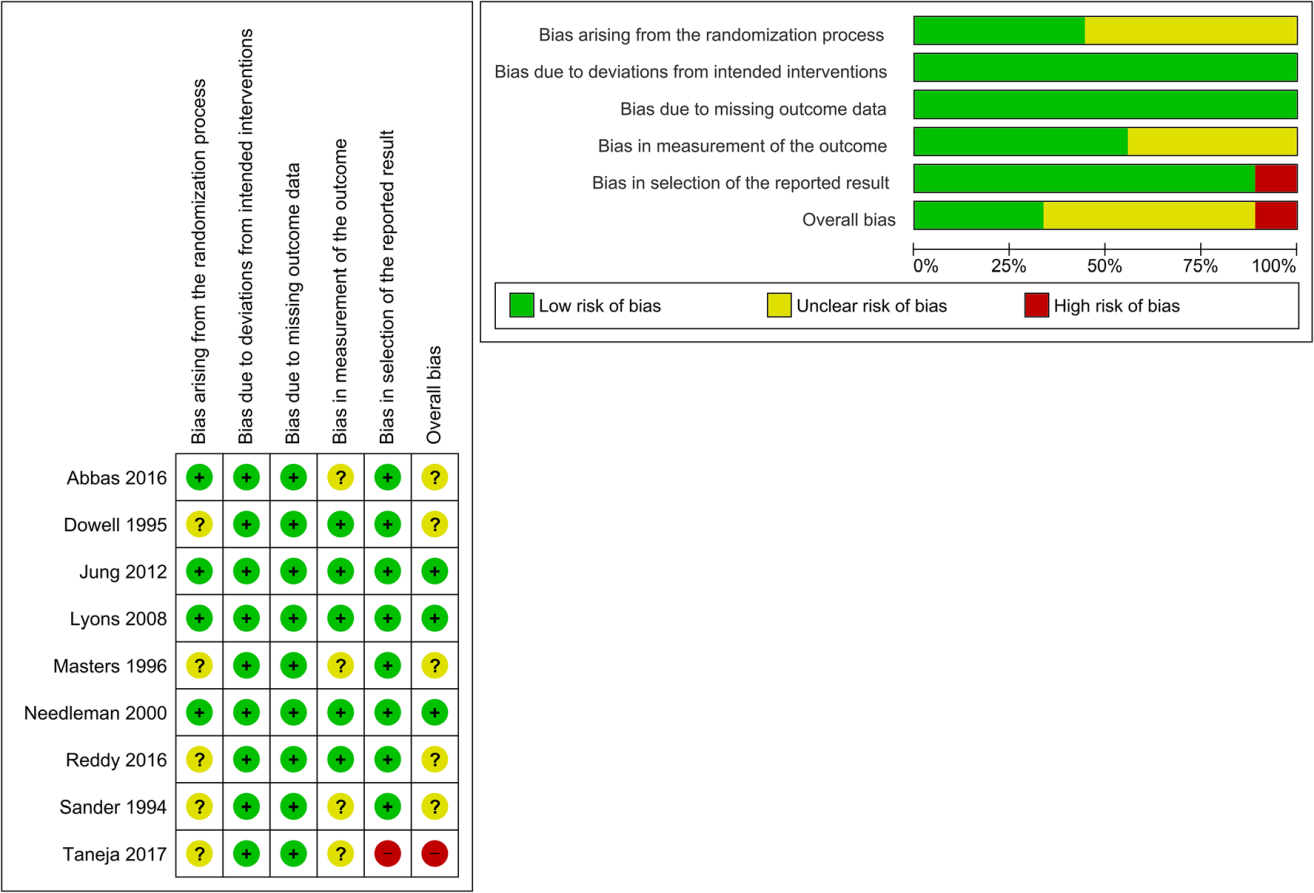
Methodological, validity and quality scores, and estimated potential risk of bias of the included studies. Risk of bias according to Cochrane Risk of Bias Tool 2 for RCTs

Out of the nine included studies, three studies (33%) were judged to have low risk of bias, five (56%) to show unclear risk, and one (11%) with high risk of bias. The most frequent source of bias was related to randomization (only 44% having low risk of bias), followed by bias in outcome measurement (56% with low risk of bias) (Figure 2).

### Meta-analysis (Figure 3)

#### CAL gain

Seven studies reported CAL gain on follow-up ≤6 months [35–41]. The antibiotic-group showed a statistically significant higher WMD CAL gain of 0.61 mm (95% CI [0.07, 1.14]; *p* = 0.03). Heterogeneity was considerable (*I*^2^ = 89%; *p* < 0.001). For effect at follow-up of >6 to 12 months, five studies reported CAL gain [33, 34, 38, 39, 41], with a statistically non-significant WMD of 0.41 mm (95% CI [−0.24,1.06]; *p* = 0.22) favoring the antibiotic-group, and substantial heterogeneity (*I*^2^ = 69%; *p* = 0.01). Sensitivity analysis could not explain heterogeneity based on variation in study design and analysis assuming a within-participant correlation *r* = 0 only made a negligible difference to the overall estimate (Appendix 4).

**Figure 3:**
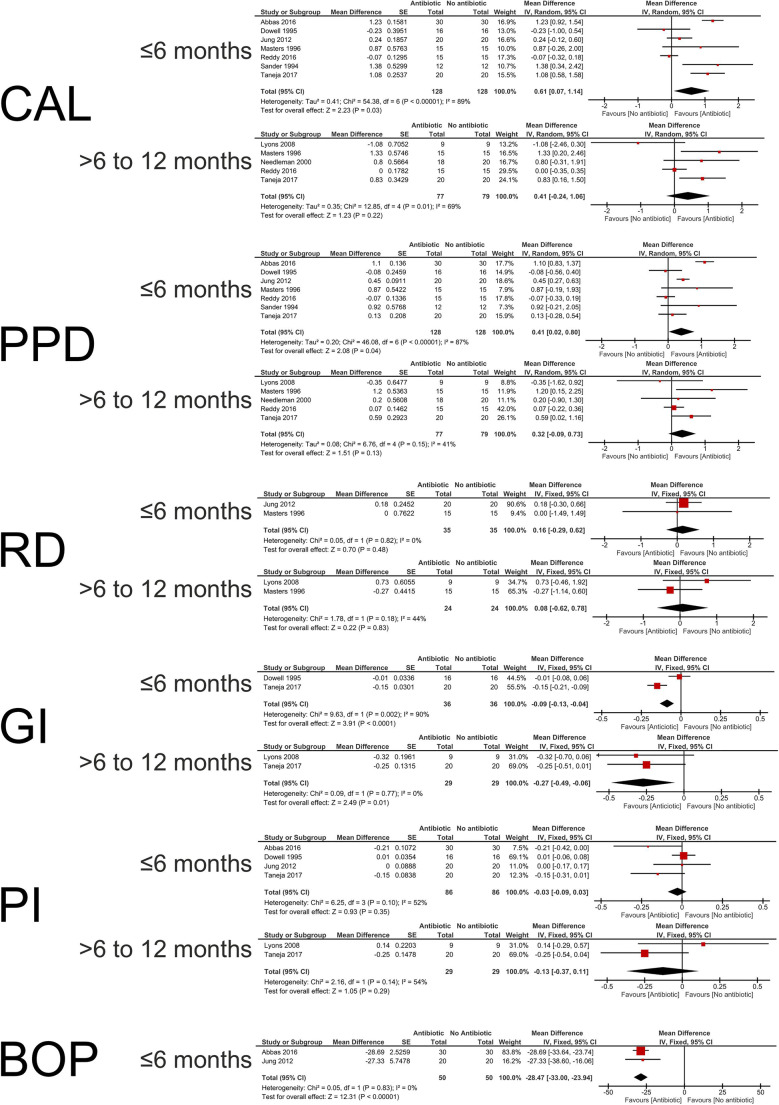
Forrest plots of the performed meta-analysis, meta-analysis for clinical attachment level (CAL) gain, probing pocket depth (PPD) reduction, recession depth (RD) changes, gingival index (GI) scores, plaque index (PI) scores, and bleeding on probing (BOP) scores for up to 6 months and more than 6 months of follow-ups for the without (control) and with (test) the adjunctive use of locally delivered antibiotics during surgical periodontal therapy. Study data, number of analyzed segments, mean difference, standard error (SE) of the difference, relative weight (%), pooled effect estimates for different outcomes, and 95% confidence intervals (95% CI) (bold) from random- and fixed-effect meta-analysis are presented. Heterogeneity was assessed by chi-square test and the *I*^2^-statistic. Z overall test statistics, *p* level of significance

#### PPD reduction

For PPD reduction, seven [35–41] and five studies [33, 34, 38, 39, 41] were included in the ≤6 months and >6 to 12 months of follow-ups’ pooled data analysis respectively. For the follow-up of ≤6 months, the antibiotic-group showed a statistically significant higher WMD PPD reduction of 0.41 mm (95% CI [0.02, 0.80]; *p* = 0.04). Heterogeneity was considerable (*I*^2^ = 87%; *p* < 0.001). For follow-up of >6 to 12 months, a statistically non-significant WMD of 0.32 mm (95% CI [−0.09, 0.73]; *p* = 0.13) favored the antibiotic-group, and heterogeneity was moderate (*I*^2^ = 41%; *p* = 0.15). Sensitivity analysis could not explain heterogeneity based on the study design, and analysis with correlation *r* = 0 only made a negligible difference to the overall estimate (Appendix 4).

#### RD changes

RD changes for follow-up of ≤6 months were reported in two studies [37, 38], while longer follow-up (>6 to 12 months) data were reported in two studies [33, 38]. WMD showed a non-significant difference regarding RD changes in both analyses, 0.16 mm (95% CI [−0.29, 0.62]; *p* = 0.48) and 0.08 mm (95% CI [−0.62, 0.78]; *p* = 0.83) respectively. Heterogeneity was low (*I*^2^ = 0%; *p* = 0.82) and moderate (*I*^2^ = 44%; *p* = 0.18) respectively.

#### GI

Two studies reported GI scores for follow-up at ≤6 months [36, 41], with the antibiotic-group showing lower GI scores with a WMD of −0.09 (95% CI [−0.13, −0.04]; *p* < 0.001) and considerable heterogeneity (*I*^2^ = 90%; *p* = 0.002). In addition, two studies reported GI-data for longer (>6 to 12 months) follow-up [33, 41], and the WMD of −0.27 (95% CI [−0.49, −0.06]; *p* = 0.01) was significantly lower in the antibiotic-group with low heterogeneity (*I*^2^ = 0%; *p* = 0.77).

#### BOP

Two studies reported BOP scores for the follow-up at ≤6 months [35, 37]. The WMD of BOP scores were −28.47% (95% CI [−33.00, −23.94]), significantly favoring the antibiotic-group (*p* < 0.001) with low heterogeneity (*I*^2^ = 0%; *p* = 0.83). Needleman et al., 2000 [34] reported that three out of 18 sites in the antibiotic-group while four of 20 sites in the control-group showed BOP. Sander et al., 1994 [40] reported that none of the surfaces in the antibiotic-group showed BOP at the follow-up, but 8.3% of the surfaces in the control-group had BOP. Due to the difference in outcome reporting the two latter studies were not included in the meta-analysis.

#### PI

Four studies reported PI-data for follow-up at ≤6 months [35–37, 41]; with a non-significant WMD of −0.03 (95% CI [−0.09, 0.03]; *p* = 0.35) and moderate heterogeneity (*I*^2^ = 52%; *p* = 0.10). Two further studies reported PI for >6 to 12 months of follow-up [33, 41] with a non-significantly lower WMD of −0.13 (95% CI [−0.37, 0.11]; *p* = 0.29) in the antibiotic-group and moderate heterogeneity (*I*^2^ = 54%; *p* = 0.14). Data from the study of Sander et al., 1994 [40] could not be included in the calculation since it only reported the percentage of surfaces showing plaque scores 2 or 3. Similarly, data from Needleman et al., 2000 reported plaque as a dichotomous outcome (yes/no: 2/18 sites in test and 6/20 sites in control) and could not be included in the meta-analysis.

#### Radiographic changes

Three studies reported radiographic outcomes, but meta-analysis was not feasible due to heterogeneity in outcome measurements. In one study [38], radiographic density values were comparable in both antibiotic- and control-groups. In the second study [39], the percentage of radiographic defect fill revealed a statistically non-significant difference, where the antibiotic-group demonstrated a fill of 31.47 ± 6.46% while the control-group 29.15 ± 6.33%. Finally, the mean radiographic increase in alveolar bone height was significantly higher in the antibiotic-group, showing a median increase of 39%, compared to 28% in the control-group in the third study [40].

### Grading the “body of evidence”

Table 2 shows the summary for various criteria used to assess the quality of evidence for the pooled estimate for CAL gain on follow-up for >6 to 12 months. Overall, evidence was rated as “very low”, being downgraded for including studies with some concerns or high risk of bias (67% of included studies), for inconsistency due to unexplained heterogeneity, and for imprecision of the treatment effect.

**Table 2:** Estimated evidence profile and appraisal of the strength of the recommendation regarding the adjunctive effect of locally delivered antibiotics during periodontal surgery on clinical attachment level gain (>6 to 12 months; primary outcome)

GRADE criteria	Rating
Risk of bias	Serious concern
Inconsistency	Serious concern
Indirectness	No (clinical outcome)
Imprecision	Yes
Publication bias	No clear bias
Other considerations for upgrading the overall rating (magnitude of the effect, dose-dependent relationship, confounders)	No
Strength of the recommendation based on the quality and body of evidence	**Very low**

## Discussion

Due to the remarkable antimicrobial activity with allegedly limited systemic side-effects, local antibiotic delivery has been suggested as a method of enhancing clinical outcomes of periodontal therapy. The present systematic review aimed to assess the effect of locally delivered antibiotics during surgical periodontal therapy on CAL gain, PPD reduction, RD changes, BOP scores, GI, PI. and radiographic defect fill. To the best of our knowledge, this is the first review assessing the possible additive benefits of locally delivered antibiotics into the periodontal defects during surgical periodontal therapy of periodontitis induced-defects.

Residual periodontal pockets of 5 mm or more are generally associated with increased risk of periodontal disease progression and tooth loss [42]. Although according to the recent guidelines of the European Federation of Periodontology (EFP), 6 mm was defined as the cut-point for performing periodontal surgery [12], the present review, in line with previous ones [43, 44], still included studies applying periodontal surgical therapy of residual periodontal pockets of 5 mm or more. The rational was to reasonably include the maximum number of RCTs addressing the topic for further strengthening the evidence of the meta-analysis. Although the current review identified a plausible number of nine eligible studies to be included, interpretation of the current review’s results should be done with caution and balanced according to the quality and number of included investigations. The nine investigations included 184 participants with 312 analyzed segments in both groups. Although all experiments were RCTs, three studies showed low [33, 34, 37], five unclear [35, 36, 38–40] and one high risk [41] of bias. Heterogenity was further notable, regarding study designs (split-mouth or parallel), blinding, number, age, and gender of participants, diagnosis of the periodontal condition requiring a surgical intervention, surgical techniques employed, antibiotic agents, their concentrations and delivery methods, and follow-up periods, with the maximum follow-up period being only 12 months in length. Seven out of nine studies were designed as split-mouth interventions, a design that could pose the risk of “carry-across effect”, where drugs delivered at one side could—through the systemic circulation or local diffusion—affect the control site and decrease the observed differences between both control and intervention groups [26, 45]. As none of the included studies investigated this effect, the real effect of antibiotic delivery may actually be higher than reported in this review. Still, this methodological heterogeneity may be interpreted in favor of versatility of antibiotic application during surgical periodontal therapy. Apart from a single study [36], reporting that one participant experienced pain in the site with antibiotic administration, with five participants having pain and unpleasant taste in the control sites 1 week post-operatively, a furcation defect in the control-group failing to close at the end of the follow-up period and the tooth consequently deemed for extraction, none of the included studies reported adverse healing effects. This absence of adverse events during the healing could endorse the locally delivered antibiotics as a safer alternative to systemically administrated ones.

Apart from significantly positive effects observed with the locally delivered antibiotics on CAL gain, PPD reduction, GI, and BOP up to 6 months of follow-ups, on longer observation period of more than 6 months, differences in favor of locally delivered antibiotics were only notable for the GI scores. A recent systematic review evaluating the effect of locally delivered antimicrobials in conjunction with non-surgical periodontal therapy [9] reported similar improvements with locally delivered antibiotic application for CAL gain (0.263 mm, 95% CI [0.123; 0.403]) in short-term with no significantly different effects observed for longer-term follow-up (12–60 months) (0.09 mm, 95% CI [−0.253; 0.433]) and a significant POD reduction in short- (0.364 mm, 95% CI [0.236; 0.491]) and long-term (0.190 mm, 95% CI [0.059; 0.321]) follow-ups respectively. A similar systematic review further reported an average CAL gain of 0.27–0.3 mm (range between −0.56 and 1.09 mm) and POD reduction of 0.30–0.48 mm (range between −0.7 and 1.13 mm) with local drug delivery following subgingival mechanical debridement [10]. Yet, the magnitudes of the reported benefits of the locally delivered antimicrobials with non-surgical periodontal therapy for CAL gain and PPD reduction appear smaller than the currently observed results. A possible explanation could rely on the assumption that antibiotics locally delivered during the periodontal surgery into the periodontal defects to be covered by the mucoperiosteal flap could incorporate the antibiotics deeper into the healing tissue, allowing for a sustained antimicrobial effect during the recovery period. In contrast a locally delivered antibiotic into periodontal pockets, following subgingival debridement, depending on the agent and its formulation (solid, gel or solution), could be washed out faster by the gingival crevicular fluid’s flow, rapidly diluting its minimal inhibitory concentration and weakening its effect [8]. Yet, it remains interesting that the plaque scores in all studies were not significant between the groups, underlying the importance of self-performed oral hygiene as the most decisive factor in plaque control.

Although the present results demonstrated improvement in periodontal parameters, their clinical significance remains to be evaluated. PPD reduction of 1 mm and CAL gain of 0.5 mm have been defined to represent clinically meaningful values from the periodontal point of view [46]. In this context, WMD of CAL gain observed in the ≤6-month follow-up period (WMD = 0.61mm) would be considered minimally clinically significant. Still, these outcomes reflect surrogate parameters, which do not fully reproduce true clinical significance. Thus, further studies evaluating long-term tooth survival, cost-benefit aspects, health risks and benefits, and possible complications are required for a founded clinical decision.

Within the limitations of this systematic review and meta-analysis, the findings suggest that the application of adjunctive locally delivered antibiotics during surgical periodontal therapy could enhance CAL gain, PPD reduction, and gingival inflammation, especially ≤6 months post-surgically, with minimal if any adverse side effects. Currently, insufficient evidence exists for a clinical recommendation. Future RCTs on the adjunctive usage of antibiotic therapy in conjunction with surgical periodontal therapy need to be conducted, with focus on true endpoints (e.g., tooth survival or oral health outcomes), aiming to identify the ideal agent, its concentration, delivery method (e.g., into membranes or bone grafts), release kinetics, antimicrobial spectrum, and possible effects on the periodontal biological healing events.

## Supplementary information

ESM 1 (DOC 548 kb)
