# Supplementary material for: Effect of locally delivered adjunctive antibiotics during surgical periodontal therapy: a systematic review and meta-analysis
Source: Clin Oral Investig. 2021 Jul 20;25(9):5127–38. doi: 10.1007/s00784-021-04056-7 (PMC8370941; doi:10.1007/s00784-021-04056-7)
Supplement: Supplementary file 1 — (DOC 548 kb) [file 784_2021_4056_MOESM1_ESM.doc]

# Manuscript: “Effect of locally-delivered adjunctive antibiotics during surgical periodontal therapy: A systematic review and meta‐analysis”

UNIVERSITÄTSKLINIKUM Schleswig-Holstein, Campus Kiel

Klinik für Zahnerhaltungskunde und Parodontologie, Direktor: Prof. Dr. C. Dörfer

Arnold-Heller-Str. 3, Haus B, D-24105 Kiel

**Prof. Dr. Matthias Hannig**

Direktor

Klinik für Zahnerhaltung, Parodontologie und Präventive Zahnheilkunde,

Universität des Saarlandes

# Editor-in-Chief

Clinical Oral Investigations

UNIVERSITÄTSKLINIKUM

Schleswig-Holstein

**Campus Kiel**

Klinik für Zahnerhaltungskunde und Parodontologie

**Ass. Prof. Dr. Karim M Fawzy El-Sayed**

Oberarzt

Arnold-Heller-Str. 3, Haus 26, D-24105 Kiel

**Tel:**	0431 / 500-26210

**Fax:**	0431 / 500-26204

**E-Mail:** karim.fawzy@gmail.com

**Date:** 30.11.2026

Dear Prof. Dr. Hannig,

Please find attached the revised manuscript “Effect of locally-delivered adjunctive antibiotics during surgical periodontal therapy: A systematic review and meta‐analysis” as a submission to *Clinical Oral Investigations.* This study systematically assesses for the first time the current evidence on effects of locally-delivered antibiotics during osseous periodontal surgery, compared to periodontal surgery alone on clinical periodontal parameters. All reviewers’ comments have been meticulously addressed and all required changes applied to the revised manuscript.

# We affirm that the study is original and not presently under consideration for publication elsewhere. In consideration of the editors of *Clinical Oral Investigations* taking action in reviewing and editing this submission, the authors hereby transfer, assign or otherwise convey all copyright ownership to *Clinical Oral Investigations* in the event that such work is published in the Journal.

We hope you are interested in our study and are looking forward to hearing from you.

Yours Sincerely,

Dr. Karim Fawzy El-Sayed
